# Supplementary material for: Towards a conceptual framework for the prevention of gambling-related harms: Findings from a scoping review
Source: PLoS One. 2024 Mar 22;19(3):e0298005. doi: 10.1371/journal.pone.0298005 (PMC10959398; doi:10.1371/journal.pone.0298005)
Supplement: S1 Appendix — (DOCX) [file pone.0298005.s001.docx]

**S1 Appendix – Grey Literature**

Sources which are highlighted in **BOLD** signify reports which were downloaded from the related website and screened for eligibility. Non-bold signifies that an organisation was searched for relevant literature, but nothing potentially relevant was found.

**Gambling**

Advisory Board for Safer Gambling

Alberta Gambling Research Institute

Australian Gambling Research Centre

Bramley S, Norrie C, Lipman V, Manthorpe J. Exploring “what works” in creating infrastructures and engagement methods for people with lived experience within public health, health and social care, and addiction research. 2020 [cited 4 November 2022]. **NIHR Policy Research Unit.** Available from https://www.begambleaware.org/sites/default/files/2020-12/final-lived-experience-infrastructure-and-engagement-project-062020.pdf.

Browne M, Bellringer M, Greer N, Kolandai-Matchett K, Rawat V, Langham E, Rockloff M, Palmer du Preez, K, Abbott M. Measuring the Burden of Gambling Harm in New Zealand. 2017 [cited 4 November 2022]. **New Zealand Ministry of Health**. Available from https://www.health.govt.nz/system/files/documents/publications/measuring_the_burden_of_gambling_harm_in_new_zealand.pdf.

Child Family Community Australia

Citizens Advice Bureau

Gam Care

Gam-Anon

Gamble Aware InfoHub

Gamblers Anonymous

GambLib (Gambling Research Library)

Gambling and Addictions Research Centre

Gambling Commission New Zealand

**Gambling Commission.** National Strategy to Reduce Gambling Harms. 2019 [cited 4 November 2022]. Available from <https://www.gamblingcommission.gov.uk/strategy/national-strategy-to-reduce-gambling-harms>.

**Gambling Related Harm All Party Parliamentary Group**. Online Gambling Harm Inquiry: Final Report. 2020 [cited 4 November 2022]. Available from <http://www.grh-appg.com/wp-content/uploads/2020/12/Online-report-Final-June162020.pdf>.

Hilbrecht M. Prevent and Education Evidence Review: Gambling-Related Harm. Report prepared in support of the National Strategy to Reduce Gambling Harms in Great Britain. 2021 [cited 4 November 2022]. **Gambling Research Exchange Ontario**. Available from <https://www.greo.ca/Modules/EvidenceCentre/files/Greo_PE-Review_Sept16-2021_FullReport.pdf>.

International Centre for Youth Gambling Problems and High-Risk Behaviours

National Problem Gambling Clinic

Open Grey

PHE - Harms associated with gambling An abbreviated systematic review

Problem Gambling Foundation of New Zealand

Problem Gambling, Wigan Council

Responsible Gambling Council

Victorian Responsible Gambling Foundation

Wardle H, Reith G, Best D, McDaid D, Platt S. Measuring Gambling-Related Harms: A framework for action. 2018 [cited 4 November 2022]. **Gambling Commission**. Available from https://assets.ctfassets.net/j16ev64qyf6l/5tpgsNwwUmqWzDEmvd2jxG/666e97cbb55a13b47c17854c2426d7af/Measuring-gambling-related-harms-framework.pdf.

**HSSF Products**

Action for Children

Action on Salt

British Medical Association

Cancer Research UK

FareShare

Food & Drink Foundation

Food Policy for Canada

Food Standards Agency

Food Standards Australia & New Zealand

Grant Thornton

Health Action Campaign

**National Food Strategy.** Independent Review – Recommendations in Full. 2021 [cited 4 November 2022]. Available from https://www.nationalfoodstrategy.org/wp-content/uploads/2021/10/25585_1669_NFS_The_Plan_July21_S12_New-1.pdf.

National Obesity Observatory

**Obesity Action Scotland.** Briefing: Advertising, Marketing and Obesity. 2019 [cited 4 November 2022]. Available from <https://www.obesityactionscotland.org/media/is1cazor/adv-marketing.pdf>.

**Obesity Action Scotland.** Briefing: Obesity and Labelling. 2019 [cited 4 November 2022]. Available from <https://www.obesityactionscotland.org/media/tglla33t/obesity__labelling_b-30.pdf>.

**Obesity Action Scotland.** Briefing: Obesity and Older People. 2022 [cited 4 November 2022]. Available from <https://www.obesityactionscotland.org/media/ubkhd1mx/obesity-and-older-people-1-final.pdf>.

**Obesity Action Scotland**. School Meals: Transforming a Feeding Culture into an Eating Culture. 2016 [cited 4 November 2022]. Available from https://www.obesityactionscotland.org/media/arcff3bj/school-mealstransforming-a-feeding-culture-into-an-eating-culture.pdf.

**Obesity Action Scotland.** Support for Healthy Weight Policies – Polling Results. 2022 [cited 4 November 2022]. Available from https://www.obesityactionscotland.org/media/qlufvkum/policy-polling-august-2022.pdf.

RAND Europe: Food Consumption in the UK: Trends, attitudes and drivers

**The Food Foundation.** The Broken Plate 2020: The State of the Nation’s Food System. 2020 [cited 4 November 2022]. Available from https://foodfoundation.org.uk/sites/default/files/2021-10/FF-Broken-Plate-2020-DIGITAL-FULL.pdf.

USFDA

WBCSD

**Tobacco**

Action on Smoking and Health

British Heart Foundation

Center for Tobacco Products

**Department of Health.** Towards a Smokefree Generation: A Tobacco Control Plan for England. 2017 [cited 15 November 2022]. Available from https://assets.publishing.service.gov.uk/government/uploads/system/uploads/attachment_data/file/630217/Towards_a_Smoke_free_Generation_-_A_Tobacco_Control_Plan_for_England_2017-2022__2_.pdf.

European Network for Smoking and Tobacco Prevention

European Respiratory Society

Foundation for a Smoke-Free World.

National Centre for Smoking Cessation and Training

**National Institute for Health and Care Excellence.** Tobacco: preventing uptake, promoting quitting and treating dependence. 2021 [cited 15 November 2022]. Available from https://www.nice.org.uk/guidance/ng209/resources/tobacco-preventing-uptake-promoting-quitting-and-treating-dependence-pdf-66143723132869.

Smoke Free Brain

Smokefree Action Coalition

The Union

Truth Initiative

**World Health Organization.** European Strategy for Tobacco Control. 2002 [cited 15 November 2022]. Available from https://apps.who.int/iris/bitstream/handle/10665/347629/WHO-EURO-2002-3882-43641-61328-eng.pdf?sequence=1.

**World Health Organization.** Framework Convention on Tobacco Control. 2003 [cited 15 November 2022]. Available from https://apps.who.int/iris/bitstream/handle/10665/42811/9241591013.pdf?sequence=1.

**Alcohol**

Al-Anon

Alcohol Change UK

**Alcohol Focus Scotland.** Tackling harm from alcohol: Alcohol policy priorities for the next Parliament. 2021 [cited 7 November 2022]. Available from [low-res-4806-afs-manifesto-tackling-harm-from-alcohol.pdf (alcohol-focus-scotland.org.uk).](https://www.alcohol-focus-scotland.org.uk/media/440069/low-res-4806-afs-manifesto-tackling-harm-from-alcohol.pdf)

Alcohol Health Alliance

Alcoholics Anonymous

British Liver Trust

**Commission on Alcohol Harm**. ‘It’s everywhere’ - alcohol’s public face and private harm. No Year [cited 7 November 2022]. Available from https://ahauk.org/wp-content/uploads/2020/09/Its-Everywhere-Commission-on-Alcohol-Harm-final-report.pdf#:~:text=The%20harm%20caused%20by%20alcohol%20is%20everywhere%20in,fully%20known%20only%20to%20those%20who%20experience%20it.

Department of Health Northern Ireland

Dimova ED, Mitchell D. Rapid literature review on the impact of health messaging and product information on packaging of alcohol and other unhealthy commodities. **Alcohol Focus Scotland**. 2020 [cited 7 November 2022]. Available from https://www.alcohol-focus-scotland.org.uk/media/440032/rapid-literature-review-on-the-impact-of-health-messaging-and-product-information-on-packaging-of-alcohol-and-other-unhealthy-commodities.pdf.

Drinkaware

Drinkwise

Institute of Alcohol Studies

International Alliance for Responsible Drinking

Mind

Nacoa

National Council on Alcoholism and Drug Dependence

National Institute of Alcohol Abuse and Alcoholism

Portman Group

**Public Health England.** The Public Health Burden of Alcohol and the Effectiveness and Cost-Effectiveness of Alcohol Control Policies. 2016 [cited 7 November 2022]. Available from https://assets.publishing.service.gov.uk/government/uploads/system/uploads/attachment_data/file/733108/alcohol_public_health_burden_evidence_review_update_2018.pdf.

Research Society on Alcoholism

Royal College of Psychiatrists

The Forward Trust

UK Smart Recovery

Unison

**University of Sterling.** Health First: An evidence-based alcohol strategy for the UK. 2013 [cited 7 November 2022]. Available from https://www.stir.ac.uk/media/stirling/services/faculties/management/documents/health-first.pdf.

World Health Organization

YoungMinds
