# Supplementary material for: Towards a conceptual framework for the prevention of gambling-related harms: Findings from a scoping review
Source: PLoS One. 2024 Mar 22;19(3):e0298005. doi: 10.1371/journal.pone.0298005 (PMC10959398; doi:10.1371/journal.pone.0298005)
Supplement: Attachment — Submitted filename: Final responses.docx [file pone.0298005.s003.docx]

| Reviewer 1 | Please note, lines cited within responses relate to those within the manuscript with tracked changes removed. |
| --- | --- |
| Comment | Response |
| The authors have made an excellent job throughout the paper and in using systematic scoping review methodology. The paper is scientifically sound and strong, and I only have some minor notes the authors may want to consider. | Thank you. We are glad you enjoyed the paper, and are grateful for your timely feedback. |
| Introduction clearly justify the need for this study. There is one excellent and potential paper to add when framing the study background: Price A, Hilbrecht M, Billi R. 2021. Charting a path towards a public health approach for gambling harm prevention. Z Gesundh Wiss. 29(1):37–53. | This paper by Alex Price provides a helpful path in charting a public health approach towards the gambling harm prevention, and was in fact originally a paper we cited within the introductory section of the paper, before replacing with Thomas et al.’s paper (lines 53-57) which also provides a pathway, and was published more recently. However, we agree that including Price et al.’s paper would add further strength to the framing of the paper, and the need for a framework which maps different approaches. We have therefore included the paper in the introductory section in lines 57-60. |
| Research methods are described in sufficient detail and a scoping review methodology is a right choice here. One of the strengths of this review was that the search included grey literature, but on the other hand, their role, robustness and/or at least added benefits for this study were not discussed. | We agree that the search for grey literature was a strength of our scoping review, and we designed in the search for grey literature to explore approaches applied by relevant organisations. However, we found that the robustness of grey literature on the whole was weaker than the peer-reviewed literature we identified. Our report is therefore based on the academic literature. We agree, however, that more detail on the use of grey literature is needed, and we have added this context to the methodology section in lines 163-169. |
| I was wondering that the search strategy begun from 2005 onwards and whether the same time cut-off was used when the authors searched grey literature. The authors cite UK-post legislation 2005. However, this choice of the year (2005) remains a bit unclear for me as a reader who is not familiar with the UK legislation. | The choice of 2005 coincides with the Gambling Act which currently governs gambling in Great Britain. The Gambling Act also controls gambling in Britain as a readily available, commercial activity. We therefore chose 2005 as a date from which to explore public health approaches to harmful commercial activities as the gambling industry developed. More context towards this end has been added to the methodology section in lines 153-159. |
| I have no other comments. I wish the authors all the best with their important line of research. | Thank you again for your helpful feedback which has strengthened our paper. |

| Reviewer 2 |  |
| --- | --- |
| Comment | Response |
| Thank you for the possibility to review this interesting manuscript. The paper focuses on building a conceptual framework to assist in the prevention of gambling-related harms. Overall, such work is highly important. While gambling is increasingly understood as a public health issue, there has been a gap in our understanding regarding how the public health approach can be operationalised at different levels (treatment, prevention, policy). A conceptual framework that includes a wide understanding of harms and acknowledges the different actors in the field is both welcome and a necessary step forward. | Thank you for taking the time to review our work. We are glad that you agree with the importance of this work. We feel that such a framework can help to bridge the gap between separate public health approaches as well as different stakeholders. |
| However, having read the current manuscript, I feel that while the framework itself is relevant and its applicability is well described, there are some major shortcomings in how it was built methodologically.  The framework builds on a scoping review that appears to take different directions. The authors have reviewed existing work on frameworks on gambling harms (table 1) as well as public health strategies from gambling and other fields (table 2). Table 4 also uses some of the existing frameworks on gambling harms to build a summary. How studies were chosen for each of these different sets of data and how they relate to each other is somewhat unclear. These should be described more systematically. | Thank you for this feedback, we agree upon reflection that the difference between the texts highlighted within these tables could be made clearer. For clarity, the main scoping review is based upon the texts sought from the criteria highlighted within Table 2, with our findings highlighted in Table 3. The frameworks highlighted in Table 1 are separate to the main scoping review, and are offered as part of the introduction, highlighting the wide range of other frameworks which are proposed by other authors.  Our scoping review moves beyond the existing frameworks which are explored in Table 1, as we explored data relating to the application of – or need for – a public health approach in the gambling sector before synthesising with approaches found in other fields. We therefore sought individual, empirical studies with participants or stakeholders which either applied a public health approach to gambling, or made recommendations for a public health approach based on behaviours or wider socio-economic or commercial determinants, before synthesising with approaches from other fields to develop the core of our framework. Table 4 relates to the categorisations of harms which we identified as part of our framework development phase. The works cited in this table, whilst they were not identified as part of the scoping review, were an important part of the categorisation of harms which were applied to our framework.  We have added extra context for clarity as to how these texts were used within the Introductory section (lines 112-119), within the methodological section (lines 183-188), as well as within Table 2 (lines 205-206). |
| The identified public health strategies in gambling and other fields (table 2) also needs more elaboration. The included gambling literature is quite limited. Furthermore, there is no discussion on how gambling actually relates to or differs from the other included fields (alcohol, tobacco, HFSS). While some lessons could probably be learnt from these sectors, they also differ from gambling in many respects. Notably, digitalisation has impacted gambling in a very different way than these substance-based issues. This leads me to question whether there could also be other public health strategies in the gambling field or whether the framework gives too much important to some strategies that are not so relevant for gambling. | Thank you for this helpful feedback. We acknowledge that our search terms may have meant that other studies which explicitly applied a public health approach to gambling may not have been returned within our scoping review. We have therefore amended the introduction to our framework in lines 325-326 as well as our conclusion in lines 505-507 to acknowledge this limitation.  We highlight other academic works which compare gambling to the alcohol, tobacco, and HSSF sectors in lines 101-107, and have highlighted how a comparison is warranted given these sectors are all commercially and legally available. Our paper builds upon these works by not only comparing approaches between sectors, but also by drawing a broad categorisation of approaches which allows the formation of a conceptual, collaborative framework inspired by lessons learned from all sectors. Digitalisation has impacted gambling through the development of new digital products which can also appear within land-based settings. This is highlighted by the categorisation of the Understanding of Environment and Product. For example, Maltzahn et al.’s study underlines the need for the specific regulation of digital products, and to specifically delink them from land-based forms of gambling such as bingo. This management of digital products could also occur alongside the management of marketing which – as highlighted in the HSSF sector – is needed to protect children. We have nonetheless acknowledged the differences between these sectors in lines 324-325.  To this end, we acknowledge that this framework is not to evaluate the strength of evidence which we have found during our scoping review, nor do we aim to propose an exhaustive list of strategies. Rather, it is to allow different stakeholders to map or evaluate extant evidence or strategies, in order to best target required interventions at the appropriate level of society. We have also acknowledged this within Lines 322-333. |
| The summary of harms (table 4) adopts the categorisation by Wardle et al. The justification that is given to this choice is that the categorisation is concise. However, is there a risk that such an approach can also neglect some important harm categories? The exclusion of crime is particularly unfortunate since as it stands, the model does not include harms that relate to the provision of gambling rather than the activity of gambling. | We acknowledge the reviewers’ comments relating crime to our model. Crime is, in fact, included within Wardle et al.’s categorisations of harm, but we have added their justification to classify crime as a resource-based harm in lines 383-388, where we highlight our decision-making process in relation to the incorporation of harm-related outcomes. We also highlight that the relationship between gambling behaviours and harms can be complex, with the possibility of categories of harms also acting as determinants behind gambling behaviours.    In relation to the exclusion of crime from the scoping review itself, our justification is highlighted in the Introduction in lines 115-116 and during the Methodology in lines 132-135. Crime was excluded from the scoping review as we felt it was more effective to produce a framework based on commercially available, legal, but potentially harmful industries. Within these commercial and legal settings, gambling itself would not be a crime. Any crime which would emerge from gambling would be classed as a resource-based harm, as per Wardle et al.’s framework. |
| The socio-ecological approach has been applied in a very similar way to gambling harms in the Wardle et al., framework. This should be acknowledged. | Thank you to the reviewer for this point. We have now acknowledged this link in the introduction in lines 116-119, and in lines 349-350. |
| On p. 33 the authors note that they prefer language that is "less emotional" than for example "severity". Severity is a terms often used to describe the degree of gambling harms. I don't see how it is emotional. | Upon reflection, we agree that severity is already an objective term. We have amended Figure 3, Figure 4, and the text on lines 394-400 accordingly. |
| There would be room for more concise language in many parts of the paper. | We have polished the language through additional readings of the paper. We would like to thank Reviewer 2 again for their helpful and timely feedback. |
