# Supplementary material for: Towards a conceptual framework for the prevention of gambling-related harms: Findings from a scoping review
Source: PLoS One. 2024 Mar 22;19(3):e0298005. doi: 10.1371/journal.pone.0298005 (PMC10959398; doi:10.1371/journal.pone.0298005)
Supplement: Attachment — Submitted filename: Responses_Tabularised_R2_Final.docx [file pone.0298005.s004.docx]

| Reviewer 2 Comments | Response (reference to lines in the version with tracked changes). |
| --- | --- |
| Thank you again for this paper. I have re-read it with a lot of interest, and I think it has improved since the last version. The authors' responses were also very helpful to me as I was trying to understand some of the methodological choices made in the paper.  I still have some comments, that partly relate to my original comments. | Thank you again for taking the time to re-read our paper. We agree that it has improved since the last version, again thanks to the helpful feedback and comments from yourself and the other reviewer. We are pleased to hear that our responses helped to make our methodological choices clear. Please find attached our responses to your comments below. |
| First, Although you explained this to me well in the response, I think the article is still somewhat unclear about where the reviews included in table 1 (and 4) are coming from. I understand from your response to my previous comments that these tables were drawn separately from the systematic review. I think the confusion stems from the fact that this is a systematic review study, and such studies have a standard practice of reporting included studies in tables that look very much like table 1 (and 4).There is need for more clarity particularly on: - How these frameworks were identified, based on what they were included in the table? - What were the inclusion criteria for including some of these frameworks in table 4 later in the paper? I think these issues could be explained under a separate heading in the methods section. | We agree with you that the focus on the framework development process within our paper could be clearer. Accordingly, we first elaborated on this process within the methods section as you recommended. However, we felt that the detail inserted here disrupted the flow of the paper, particularly as we outlined the methods for our scoping review. We have added this information in lines 338-350, where we feel that it adds context to the development of our framework. |
| Second, I am still slightly stuck on the issue of crime. I appreciate the explanation given by the authors that crime can relate to the illegal provision of gambling, and was therefore excluded. This was mentioned at least in three different parts of the manuscript: p.5.line 117; p. 10 line 136->; page 34. (version with track changes). In the response to my previous comments, the authors note that crime that emerges from gambling is still included as a subcategory of harms. I think this needs to be in the manuscript as well. Furthermore, the paper by Banks & Waugh (that the authors also reference) also suggests that there are categories of gambling-related crime that are for example compliance-related. Are these included? I think just a clarifying sentence here would be enough. | You have raised a good point on the nature of crime as a GRH-related outcome. We have added context in Lines 424-428, explicitly stating that crime is included as a GRH-related outcome, with crime including activity defined by Wardle et al., as well as industry-based, compliance-related crimes as highlighted by Banks and Waugh. |
| Third, you note on page 11 line 173 (version with track changes) that you only based the analysis on peer reviewed literature and not grey literature. In the table with the included studies, you have also included some opinion pieces or correspondence. Are these peer reviewed? | These papers are peer-reviewed. This has been added for clarity in line 326. |
| Fourth, this is a suggestion: P. 31 has good discussion on the differences between gambling and substance-based commodities. You might add in a sentence or two summarising the comparison of the PH strategies identified across gambling/ other sectors. Were there strategies that were not captured by the gambling literature but that were included in the literature on the other commodities? Which ones were they and how did the emphasis differ across these products? | Thank you for this suggestion. We have added some further, brief discussion in lines 354-359 that provides an overview of the strategies uncovered across sectors. |
|  | Thank you again for your comments and feedback on both rounds of revision. We are grateful to your help in strengthening our paper. |

| Reviewer 3 |  |
| --- | --- |
| Reviewer #3: This is an excellent paper on a very important topic. This conceptual framework is highly needed in the sector as it will serve the needs of multiple stakeholders and support the development and implementation of various initiatives. The authors have satisfactorily addressed the previous reviewers' comments. Only two small suggestions for consideration: | Thank you for your feedback and suggestions. We are glad you enjoyed the paper, and we are pleased to hear you agree that the framework is needed. Please find our response to your suggestions detailed below. |
| 1. In the introduction, lines 35-36, the authors refer to "other harmful but legal sectors." Perhaps the authors want to reword so as to not label the other sectors as "harmful?" They instead may want to consider language such as "other sectors focused on reducing harms." | Thank you for this suggestion. After careful consideration, we have decided to amend the wording to “potentially harmful”. “Harmful” is a term used elsewhere within the literature we have cited (for example, see ref 40, Thomas et al.). Additionally, the term “other sectors focused on reducing harms” implies that these sectors are wholly focused on the reduction of harms. While our paper draws upon *research* seeking to reduce harms within those sectors, we cannot conclude that the industries themselves are actively seeking to reduce harms. Conversely, literature that we cite (see refs 40-41) highlight that industry actors act in self-interest, regardless of the harms caused. |
| 2. In a couple places the authors use the phrase "thanks to" in regards to digital transformation. They authors may want to consider "in part due to" instead. | We have removed “thanks to” and have added “due to” in line 29 and line 46. To add further clarity, we have also added “liberal deregulation” to highlight its importance alongside digital transformation. |
|  | Thank you again for your helpful comments which have strengthened our paper. |
